# Comprehensive nursing model for diabetic foot ulcers: A strategy to improve prognosis and quality of life

**DOI:** 10.1097/MD.0000000000038674

**Published:** 2024-06-28

**Authors:** Jingjing Zhou, Lili Zhou

**Affiliations:** a Department of Endocrinology, The Second Affiliated Hospital of Harbin Medical University, Harbin, China; b Department of Nephrology, The Second Affiliated Hospital of Harbin Medical University, Harbin, China.

**Keywords:** amputation rate, comprehensive nursing, diabetic foot, diabetic foot ulcer

## Abstract

Diabetic foot (DF) ulcer is one of the common complications of diabetic patients, with high incidence and amputation rate, which seriously affects the quality of life and health of patients. Therefore, how to effectively prevent and treat DF ulcers and reduce amputation rate has become an urgent problem in the medical field. As a comprehensive nursing model for patients with DF ulcers, comprehensive nursing intervention is designed to improve the therapeutic effect and prognosis and reduce the rate of amputation. Convenient sampling method was used to select 360 patients with DF who received routine care for DF ulcers from July 2013 to July 2023 for retrospective cohort analysis. According to the existence of exposure factors (comprehensive nursing intervention), 180 cases were divided into observation group and comparison group. The basic demographic data, amputation rate, severity of foot ulcer, neuropathy and vascular disease, and blood glucose control were compared between the 2 groups. The data was analyzed using SPSS26.0. Harman single factor test was used to check whether there was common method bias in the study data. Descriptive analysis, Spearman rank correlation analysis and multiple linear regression analysis were used to analyze the current situation of amputation rate of DF patients and the influence of comprehensive nursing intervention on the amputation rate of DF patients. The amputation rate was 2.8% in the Observation group compared to 8.3% in the Comparison group. The amputation rate of the observation group was generally higher in the age group, and the amputation rate of the observation group was higher in the middle school education level and below and the economic status of <5000 yuan. The difference was statistically significant (*P* < .05). Age (odds ratio [OR] = 1.96; 95% confidence interval [CI]: 0.88–4.38), education level (OR = 1.30; 95% CI: 1.69–6.46), economic status (OR = 2.28; 95% CI: 1.69–10.85) was an independent risk factor for amputation rate (*P* < .05). Comprehensive nursing interventions have played a positive role in reducing the rate of amputation in patients with DF.

## 1. Introduction

Diabetic foot (DF), one of the common chronic complications of diabetes, refers to foot infection, ulceration, and deep tissue destruction caused by local nerve abnormalities and peripheral vascular diseases of the lower extremity.^[1]^ Patients with diabetes who have had the disease for >5 years or who have poorly controlled blood sugar levels are at an increased risk for developing severe DF complications, including infections, ulcers, and gangrene.^[2]^ According to statistical data, in Western countries, the annual economic cost associated with DF ulcers ranges from approximately 16,000 to 27,000 US dollars, with the medical expenses for amputation being even higher at around 43,000 to 64,000 US dollars per year. These costs primarily cover expenses related to home care and social services. Failure to properly manage DF wounds can result in local or systemic infections, with severe cases potentially leading to gangrene and necessitating amputation. Diabetic patients face a significantly higher risk of amputation compared to non-diabetic individuals, with studies indicating that 70% of amputations occur in diabetic patients.^[3]^ The DF presents distinct characteristics from typical chronic trauma, exhibiting a slow progression and challenging healing process influenced by various factors including comorbidities. The substantial economic and psychological burdens associated with long-term management pose significant challenges for affected families. Consequently, early intervention and comprehensive nursing care for diabetic patients are crucial.

The long-term prevention and treatment of DF is particularly important in the comprehensive nursing management of DF. Ahmad et al’s^[4]^ research findings indicate that a significant proportion (9.85%) of amputations related to DF complications could potentially be prevented through proactive and efficacious interventions. These results underscore the importance of implementing aggressive prevention and treatment strategies to mitigate the incidence and rate of amputations associated with DF. Additionally, the implementation of standardized and comprehensive nursing management practices is crucial in effectively preventing and treating DF complications, thereby reducing the overall amputation rate attributed to this condition.^[5]^ Comprehensive nursing intervention is a comprehensive and multidisciplinary nursing method, including health education, diet guidance, exercise intervention, drug therapy, and other aspects of nursing. Through comprehensive nursing intervention, patients’ understanding of DF and self-management ability can be improved, so as to better control the disease and reduce the occurrence and amputation rate of DF. Gypdonck et al’s^[6]^ research indicates that nursing interventions have the potential to enhance the understanding of leg care among DF patients, leading to improved adherence to nurses’ recommendations regarding healthy lifestyle practices. This finding underscores the importance of nursing interventions in empowering patients to comprehend their condition and embrace proactive measures for its management. Barshes et al’s^[7]^ studies have found that the incidence of amputations in the United States is closely related to DF. Although the prevention and management strategies for DF are constantly developing, lack of comprehensive care is still one of the obstacles to effective prevention of DF.^[8]^ These results indicate that although there are many prevention and management strategies for DF that have been developed, it is still difficult to effectively prevent the occurrence and amputation of DF without the implementation of comprehensive nursing interventions.

Comprehensive nursing intervention plays an important role in the prevention and treatment of DF. Through the implementation of comprehensive nursing intervention, patients can improve their understanding of DF and self-management ability, reduce the incidence of DF and amputation rate, and improve the quality of life of patients. Hence, it is imperative to enhance comprehensive nursing interventions in order to enhance patients’ adherence and self-management skills, thereby improving their ability to control DF conditions and reducing the incidence of amputations. Consequently, comprehensive nursing intervention is essential in the prevention and management of DF. Therefore, we selected DF patients as the study objects to explore the effect of comprehensive nursing intervention on reducing the amputation rate of DF ulcers.

## 2. Patients and methods

### 2.1. Comparison of general information

A retrospective cohort analysis of 360 patients with DF who received routine care for DF ulcers from July 2013 to July 2023 was conducted using convenient sampling method. According to the presence or absence of exposure factors (comprehensive nursing intervention), 180 patients were divided into observation group and control group. This study has been approved by the Ethics Committee of the Second Affiliated Hospital of Harbin Medical University. We hid personal patient information when including study data.

#### 2.1.1. Inclusion criteria

Complete clinical data of patients. Meets Wagner criteria for DF levels 0 to 4. There are obvious foot problems, such as calluses, gray nails, corns, inlay nails, etc. With clear awareness and strong communication skills, questionnaires can be filled out on their own or with the help of family members and investigators. Patients volunteered for the survey.

#### 2.1.2. Exclusion criteria

Patients with non-diabetic skin rupture in any part of the foot and/or lower extremities. Patients with cognitive and communication impairments. Lower extremity arteriovenous color ultrasound showed complete arteriovenous occlusion. I don’t want to take part in this survey.

### 2.2. Nursing methods

All DF patients included in this study had a history of foot ulcer, and the intervention time of all patients was once a day before discharge, once every month after discharge, and continued intervention for 6 months. Assess the patient at the end of the intervention. Patients in both groups received routine care for DF ulcers and diet control: Healthcare providers should educate patients and their families on the significance of adhering to dietary restrictions in the management of diabetes and DF conditions, while also promoting active participation and adherence. Individualized dietary plans should be developed by nursing staff based on factors such as the patient’s weight, blood sugar levels, exercise regimen, and eating habits. These plans should prioritize controlling overall calorie intake and ensuring a well-balanced nutritional profile. The recommended nutrient distribution for each meal should consist of approximately 50 to 60% carbohydrates, 20 to 25% protein, and 20 to 25% fat. The caloric distribution of 3 meals a day should be reasonable, of which breakfast, lunch, and dinner account for 1/5, 2/5, and 2/5 respectively. Patients should abstain from tobacco, alcohol, and spicy food.

#### 2.2.1. Exercise instruction

Patients should be instructed daily to exercise with the Bigle exercise method to effectively improve the blood circulation of the affected limb. The patient should lie flat on the bed, raise the affected limb 45° 2 minutes, put it flat, then raise it, put it flat, and repeat 8 to 10 times; The affected foot moves up, down, left, and right in different directions, twice/d, 5 to 10 minutes each time. In cases where patients are immobile due to severe ulcers, it is recommended that nursing staff aid in moving the affected limb at least twice daily, provide regular massages to the pressured area, and instruct patients in performing foot lifting exercises at the bedside for 5 to 10 minutes per session. As the ulcer approaches healing, patients may be encouraged to stand with the support of a chair for leg-shaking exercises and gradually progress to walking. It is important that the level of activity be increased gradually and not excessively.

#### 2.2.2. Medication care

Patients must adhere to their physician’s recommendations regarding the administration of oral hypoglycemic medications, refraining from altering dosage or discontinuing treatment. It is imperative to consume a meal within 30 minutes following subcutaneous insulin injection in order to maintain consistency in insulin onset and postprandial blood sugar elevation, thereby minimizing the risk of hypoglycemic reactions. Patients and their caregivers should be educated on how to recognize and prevent hypoglycemic episodes. Regular monitoring of blood glucose levels and urine sugar is essential to facilitate dosage adjustments of oral hypoglycemic drugs and insulin by healthcare providers.

#### 2.2.3. Foot care

Patients should maintain proper foot hygiene by keeping their feet clean and dry, wearing cotton, soft, and loose shoes, and socks, avoiding trimming toenails too short, and refraining from scratching the skin of their feet. It is important to keep feet warm to prevent frostbite or burns and to avoid prolonged walking or sitting in a cross-legged position to prevent impairment of lower limb blood circulation. Patients should be educated on the benefits of foot massage, recommending a frequency of 1 to 2 times daily for 15 to 20 minutes each session to enhance blood circulation and facilitate recovery from the disease.

#### 2.2.4. Ulcer wound treatment

In cases of clean wounds, a mixture of 654 to 210 mg can be combined with 8 U of ordinary insulin and applied locally with a wet compress. If there are excessive wound secretions, the wound can be alternatively washed with 3% hydrogen peroxide and normal saline before applying a mixture of gentamicin (80,000–240,000 U), ordinary insulin (8–20 U), 654 to 210 mg, and normal saline with a wet compress and sterile gauze bandage. For deep wounds with significant pus and necrotic tissue, the necrotic tissue should be removed aseptically or through incision and drainage prior to the aforementioned treatment regimen, with adjustments made as necessary based on the specific circumstances.

The observation group received comprehensive nursing intervention. Diet control: For people with diabetes, diet control is key. High blood sugar can cause foot ulcers to worsen, so patients should understand and follow healthy eating habits. According to the patient’s weight, blood sugar value, exercise and eating habits, and other factors, nursing staff can develop scientific and reasonable personalized recipes for them. The main principle of the diet should be to control the total calorie intake and ensure balanced nutrition. For people with diabetes, they should avoid foods high in sugar, fat, and salt, minimize the intake of sugars, and eat more foods rich in fiber, such as vegetables and fruits.

#### 2.2.5. Exercise guidance

Patients should do an appropriate amount of exercise every day, such as 5 to 10 minutes, 2 times a day. For patients who are unable to move due to severe ulcers, the caregiver should assist them in moving the affected limb and regularly massage the area under pressure.

#### 2.2.6. Medication care

Eat 30 minutes after subcutaneous insulin injection to avoid hypoglycemic reaction. In addition, the injection site should also be observed for allergic reactions.

#### 2.2.7. Foot care

Patients should keep their feet clean and dry to prevent infection. Shoes and socks should be cotton, soft, and loose, avoid wearing too tight or hard shoes. Do not trim your toenails too short to avoid damaging the surrounding skin. Walking for long periods of time increases pressure on the feet while sitting cross-legged can affect blood circulation in the lower extremities. Church patients or their families for foot massage, massage 1 to 2 times a day, 15 to 20 minutes each time, can improve blood circulation, and promote the recovery of the disease.

#### 2.2.8. Ulcer wound treatment

If the wound is clean and not infected, it can be cleaned with normal saline applied with iodophor, and then wrapped with sterile dressing. If the wound is infected, anti-infection treatment should be performed first. Appropriate antibiotic drugs can be selected according to the wound condition. Applying a wet wound compress with medication can help clean the wound, reduce infection, and promote healing. Commonly used wet compress drugs include gentamicin, 654-2, and so on. If the wound is deep, there is pus or more necrotic tissue, debridement and drainage should be performed first. Necrotic tissue is removed under aseptic operation or incision and drainage, and then wet compress and other treatment as described above. Treat once or twice a day according to the situation.

### 2.3. Observation indicators and judgment criteria

Wound pain scoring: Digital grading (NRS) is a pain scoring method proposed by Burkitt et al in 1993, which includes patient’s main complaint, physical response, behavioral response, and emotional response.^[9]^ The NRS is scored on an 11-point scale, from 0 to 10, with higher numbers indicating higher pain. 0 is painless and 10 is the most intense pain. The clinical significance is to provide a simple and effective pain assessment tool for clinicians to better control patients’ pain. As for the reliability and validity of NRS score, some studies show that the score has good reliability and validity. One study showed that the NRS score was highly correlated with the visual analog scale score in assessing postoperative pain (*R* = 0.82), indicating that the NRS score can effectively assess the degree of postoperative pain. In addition, NRS score also has good retest reliability, retest reliability is 0.88, indicating that the score has good reliability.

#### 2.3.1. Measurement of wound area

Using disposable DF measurement paper, wound width measurement is the widest point parallel to the horizontal axis of the human body, length measurement is the strongest point parallel to the vertical axis of the human body, and then the wound area (cm^2^) = length (cm) × width (cm) is obtained.

### 2.4. Statistical analysis

Excel software was used to establish a database for all the patient data included in our study. After logical verification, the data were imported into Statistical Product and Service Solutions 26.0 software (IBM, Armonk) for data analysis. Harman single factor test was used to test the common method bias, and if the explanation rate of the largest common factor was <40%, it was considered that there was no serious common method bias between the study data. Counting data is expressed as integers or percentages, *x*² tests are used for comparison between groups, and rank sum tests are used for ordered variables. The measurement data were represented by mean ± SD. Descriptive statistical analysis was performed on the measurement data first, and Shapiro–Wilk method was applied for normality test. When the age of patients followed normal distribution, *t* test was applied. The Mann–Whitney *U* rank sum test was applied when the normal distribution was not met, with a *P* value < 0.05 indicating a statistically significant difference.

## 3. Results

### 3.1. Comparison of demographic characteristics and clinical data

The mean age of the observation group was 53.91 ± 3.81 years, while that of the control group was 52.97 ± 3.78 years. Statistically, the age of the observation group was higher than that of the control group, and the difference was significant (*P* = .009). The number of patients with junior high school education and below was higher in the observation group than in the control group (*P* = .003). The number of patients with economic status >5000 yuan in observation group was higher than that in control group (*P* = .036). Other clinical features such as lower extremity neuropathy, lower extremity vasculopathy, foot deformity, foot callus, foot corns, foot nail embedding, foot ringworm, foot cleft and foot blister, and short toenail clipping were not significantly different between the 2 groups (*P* > .05). See Table 1.

**Table 1:** Comparison of clinical characteristics between the 2 groups (x̅ ± SD, n).

Project	Observation group (180)	Comparison group (180)	*t*/*x*^2^	*P* value
Age (yr)	53.91 ± 3.81	52.97 ± 3.78	2.350	.009
BMI (kg/m^2^)	28.25 ± 3.58	28.35 ± 3.18	0.961	.325
Gender (male/female)	116/64	112/68	0.191	.662
Wound area (cm^2^)	26.21 ± 6.37	26.22 ± 6.36	0.014	.494
NRS score	2.83 ± 0.36	2.72 ± 0.38	0.382	.301
Fasting blood glucose	16.87 ± 2.02	16.50 ± 2.09	1.707	.044
Complication			0.245	.885
Hypertension	65	62		
Hyperlipemia	69	68		
Other	46	50		
Educational level			8.739	.003
Junior high school and below	130	153		
Above junior high school	50	27		
Marital status			1.111	.292
Be married	140	148		
Spinster	40	32		
Economic situation			4.392	.036
>5000 RMB	107	126		
<5000 RMB	73	54		
Lower extremity neuropathy			0.899	.343
Yes	150	143		
None	30	37		
Lower extremity vascular disease			0.854	.356
Yes	130	153		
None	30	27		
Foot deformity			1.143	.285
Yes	127	136		
None	53	44		
Callus of the foot			0.829	.362
Yes	120	128		
None	60	52		
Foot corns			0.766	.382
Yes	110	118		
None	70	62		
Inlaid nails on the feet			1.135	.287
Yes	108	98		
None	72	82		
Tinea pedis			1.418	.234
Yes	116	105		
None	64	75		
A tear in the foot			0.190	.663
Yes	111	115		
None	69	65		
Foot blisters			2.195	.138
Yes	91	105		
None	89	75		
Toenail clippings are too short			0.403	.525
Yes	101	95		
None	79	85		

BMI = body mass index, NRS = digital grading.

### 3.2. Comparison of amputation rates with different demographic characteristics and clinical data

The amputation rate was 2.8% in the Observation group (5 cases) and 8.3% in the Comparison group (15 cases). The comparison of amputation rate between different demographic characteristics and clinical data is as follows: the amputation rate of the observation group is generally larger in age, and the amputation rate of the observation group is higher in junior high school education or below and economic status < 5000 yuan. The difference was statistically significant (*P* < .05). The incidence of lower extremity neuropathy, lower extremity vasculopathy, foot deformity, foot callus, foot corns, foot nail embedding, tinea pedis, foot cleft, and foot blister was generally higher in the observation group. However, these differences did not reach the level of significance (*P* > .05). See Table 2.

**Table 2:** Comparison of amputation rates with different demographic characteristics and clinical data.

Project	Observation group (5)	Comparison group (15)	*t*/*x*^2^	*P* value
Age (yr)			4.444	.035
≤ 60	1	10		
> 60	4	5		
Gender (male/female)	4/1	9/6	.659	.417
Complication			.114	.944
Hypertension	2	5		
Hyperlipemia	2	6		
Other	1	4		
Educational level			4.356	.037
Junior high school and below	3	2		
Above junior high school	2	13		
Marital status			0.131	.718
Be married	4	13		
Spinster	1	2		
Economic situation			6.667	.010
>5000 RMB	3	1		
<5000 RMB	2	14		
Lower extremity neuropathy			0.131	.718
Yes	4	13		
None	1	2		
Lower extremity vascular disease			3.268	.071
Yes	3	14		
None	2	1		
Foot deformity			0.351	.554
Yes	5	14		
None	0	1		
Callus of the foot			1.667	.197
Yes	3	13		
None	2	2		
Foot corns			0.317	.573
Yes	3	11		
None	2	4		
Inlaid nails on the feet			0.000	1.000
Yes	3	9		
None	2	6		
Tinea pedis			0.800	.371
Yes	3	12		
None	2	3		
A tear in the foot			0.317	.573
Yes	3	11		
None	2	4		
Foot blisters			0.317	.573
Yes	3	11		
None	2	4		
Toenail clippings are too short			0.055	.815
Yes	1	5		
None	4	15		

### 3.3. Multiple Logistic regression analysis affecting amputation rate

The factors influencing the amputation rate were analyzed by sequential multiple Logistic regression. Whether the patient had amputation or not was taken as the dependent variable, and different demographic characteristics and clinical data with statistical significance were taken as independent variables (age: ≦60 years old = 0; >60 years old = 1. Education level: junior high school and below = 0; Above junior high school = 1. Economy: >5000 yuan = 0; <5000 yuan = 1). The results showed that age (odds ratio [OR] = 1.96; 95% confidence interval [CI]: 0.88–4.38), education level (OR = 1.30; 95% CI: 1.69–6.46), economic status (OR = 2.28; 95% CI: 1.69–10.85) was an independent risk factor for amputation rate (*P* < .05). See Table 3.

**Table 3:** Multiple Logistic regression analysis affecting amputation rates.

	*β*	SE	Wald *x*^2^	*P*	OR	95% CI
Age	0.292	0.857	7.074	.018	1.96	0.88–4.38
Educational level	0.518	0.617	7.545	.019	1.30	1.69–6.46
Economic situation	0.631	0.574	6.067	.024	2.28	1.69–10.85

CI = confidence interval, OR = odds ratio.

## 4. Discussion

Our study revealed a statistically significant difference in amputation rates between the Observation group (2.8%) and the Comparison group (8.3%), indicating that comprehensive nursing interventions may play a role in reducing amputation rates in DF patients. Furthermore, the observation that amputees in the Observation group tended to be older suggests that age may be a contributing factor to the progression of DF complications and the subsequent need for amputation. In addition, the amputation rate of the observation group was higher for those with junior high school education and below an economic status < 5000 yuan. This suggests that individuals with lower levels of education and financial resources may be at a heightened risk for DF complications and subsequent amputation. Multiple logistic regression analysis revealed age, education, and economic status as independent risk factors for amputation rates. These findings imply that, beyond medical interventions, a patient’s socioeconomic standing and educational background may play a significant role in determining the likelihood of amputation in DF patients.

In our multiple Logistic regression analysis, age, education, and economic status were identified as independent risk factors for amputation rates. Relevant research results show that the older the age, the higher the amputation rate, which may be related to the older age, poor immune ability, and poor physical condition of DF patients.^[10]^ These factors have the potential to result in patients experiencing forgetfulness or inaccurately recalling the guidance provided by healthcare professionals. Furthermore, elderly individuals may exhibit a slower rate of habit change as a result of the constraints imposed by their medical conditions, familial responsibilities, and living environment.^[11]^ Therefore, nursing interventions for older DF patients require more patience from caregivers. The higher the education level, the higher the amputation rate, which may be related to the better understanding and acceptance of knowledge of patients with higher education levels, and their ability to recognize the benefits of amputation on their own disease.^[12]^ This suggests that a patient’s awareness and understanding of amputation may affect their amputation rate.

The findings indicate a significant correlation between patients’ monthly income and the rate of amputation, suggesting that income constraints may impede patients’ ability to adhere to treatment plans or recommendations from healthcare providers. Consequently, monthly income emerges as a key determinant influencing the amputation rate among patients. Age is an important factor affecting the amputation rate of DF patients. With age, the patient’s immune capacity, and physical condition may gradually decline, and the resistance to disease may also weaken. Therefore, for older DF patients, medical staff should strengthen nursing intervention and provide more detailed and thoughtful nursing services to reduce the amputation rate of patients.^[13]^ The educational attainment of DF patients is a significant determinant of amputation rates. Those with higher levels of education demonstrate greater comprehension and acceptance of medical information, leading to a better understanding of the benefits of amputation in managing their condition. Consequently, healthcare providers should tailor health education interventions to patients’ educational and cognitive capacities to enhance disease awareness and self-management skills, ultimately reducing the incidence of amputations.^[14]^ Economic conditions are also an important factor affecting the amputation rate of DF patients. As the treatment of DF requires long-term medical expenses, some patients with poor economic conditions may not be able to bear the high medical expenses and thus cannot get timely and effective treatment.^[15]^

Hence, it is imperative for healthcare professionals to consider the financial circumstances of patients and offer them optimal assistance and support, such as aiding in the application for medical aid and providing discounted medical services, to alleviate the economic strain on patients, enhance their adherence to treatment, and improve treatment outcomes.^[16]^ Age, education level, and economic condition are the important factors affecting the amputation rate of DF patients.^[17]^ Healthcare professionals should provide individualized guidance and management tailored to the unique needs of each patient, enhance nursing interventions and health education, and enhance patients’ self-management skills in order to decrease the rate of amputations among patients.^[18]^ Simultaneously, it is imperative for both the government and society to enhance their focus and assistance towards DF patients, offer more accessible and beneficial medical services, alleviate the financial strain on patients, and enhance patient adherence to treatment regimens and overall treatment outcomes.^[19, 20]^

## 5. Conclusion

The influence factors of comprehensive nursing intervention on amputation rate of DF patients included age, education level, and monthly income level. These factors should be paid attention to in the treatment and management of DF patients. Comprehensive care interventions may include measures such as health education, dietary guidance, exercise training, and psychological support that can help patients better control blood sugar and improve their quality of life. In conclusion, comprehensive nursing intervention has a positive effect on reducing the amputation rate of DF patients, and the factors affecting the amputation rate include age, education level, and economic status. To better manage and treat people with DF, physicians, and social workers should focus on these factors and provide appropriate integrated care interventions.

## Acknowledgments

We are grateful to the Second Affiliated Hospital of Harbin Medical University for its support and assistance in the research.

## Author contributions

**Data curation:** Lili Zhou.

**Resources:** Lili Zhou.

**Writing – original draft:** Jingjing Zhou.
